# Rethinking Learning Experience: How Generally Perceived Life Stress Influences Students’ Course Perceptions in Different Learning Environments

**DOI:** 10.3390/ejihpe13080109

**Published:** 2023-08-12

**Authors:** Morris Gellisch, Thorsten Schäfer, Imadeldin Yahya, Matthias Joswig, Xin Cheng, Gabriela Morosan-Puopolo, Beate Brand-Saberi

**Affiliations:** 1 Department of Anatomy and Molecular Embryology, Institute of Anatomy, Medical Faculty, Ruhr University Bochum, 44801 Bochum, Germany; 2 Center for Digital Teaching and Learning in Medicine, Medical Faculty, Ruhr-University Bochum, 44801 Bochum, Germany; 3 Department of Anatomy, Faculty of Veterinary Medicine, University of Khartoum, Khartoum 11115, Sudan; 4 Division of Histology and Embryology, Joint Laboratory for Embryonic Development & Prenatal Medicine, Medical College, Jinan University, Guangzhou 510632, China

**Keywords:** medical education, stress and learning, digital learning environments

## Abstract

Previous research work has already demonstrated that both the form of teaching as well as different teaching methods directly influence students’ learning experience along with their psychobiological responses at the endocrine and autonomic level. Aiming to gain deeper insights into the constitution of the learning experience, this study examined the influence of external factors such as generally perceived life stress and self-efficacy on the immediate learning experience in different learning environments. Therefore, a randomized experimental field study was conducted in which both psychological constructs and physiological data (heart rate variability) were collected from healthy first-year medical students (*n* = 101) during the COVID-19 pandemic. In an effort to determine the consistency of the effects across various teaching formats, the same content of a practical histology course was carried out in a face-to-face setting as well as in passive and active online teaching. While self-efficacy was a strong predictor for positive course perceptions in all learning conditions (Pearson’s r = 0.41–0.58), generally perceived worries correlated with higher anxiety during passive online learning and face-to-face learning (Pearson’s r = 0.21–0.44), a finding supported by the negative correlation between the level of perceived life demands and enjoyment during the learning unit (Pearson’s r = −0.40–−0.43). Here, we additionally report initial evidence pointing towards the role of reduced general life stress as a resilience factor for the expression of physiological stress parameters in an academic context (small-sized effect; Pearson’s r = 0.18). The data gathered in this study illustrate the relevance of emerging emotional manifestations—either aversive; negative effect or positive; protective effect—for the immediate learning process and thus establish a connection between medical education and the importance of mental health and wellbeing—especially discussed against the background of current social and political challenges in increasingly complex societal structures.

## 1. Introduction

Students’ perception of their learning experience as well as perceived emotions in learning environments are highly relevant target constructs of educational science research, as they are closely related to learning engagement and good educational practice [1,2,3,4,5]. Further, emotions experienced in academic settings are valid predictors of students’ motivational characteristics and academic performance [6,7,8,9,10]. Pekrun et al. [11] developed and validated the Achievement Emotions Questionnaire (AEQ) to quantify perceived emotions in academic settings and were able to demonstrate that the valence of an emotion—positively toned; enjoyment/negatively toned; anxiety—is of fundamental importance for academic achievement, cognitive resources, and motivation [12].

It has already been shown that activating teaching methods and increased interaction during a learning unit strongly influence students’ learning experience [13,14,15]. By developing an appropriate work and learning environment, the lecturer is thus able to modulate the learning experience of students participating in the respective course [16]. However, since it could also be shown that environmental factors and the state of health of students influence academic performance [17], the research question arises as to whether and to what extent factors external to the learning unit influence students’ learning experience. This overarching question takes on a special significance against the background of current global changes, causing psychosocial alterations, not least due to the particularly demanding pandemic situation and further complex societal challenges such as dealing with climate change, natural disasters, and Russia’s war of aggression in Ukraine.

One factor of substantial importance is perceived life stress, as it exerts an enormous impact on general well-being, serves as a reliable predictor for exhaustion, and is also linked to various serious illnesses such as depression, cardiovascular diseases, and immune dysfunction [18,19,20,21]. A factor associated with similarly aversive components is social anxiety, which—based on social–evaluative situations—is directly related to pervasive fear and avoidance [22,23,24]. Since it has also been shown that social anxiety is related to impairments in attention and academic achievement [25,26], the question then arises as to the influence of social anxiety on concrete learning experiences in different learning environments.

Acutely aversive, emotionally stimulating, or demanding situations can also manifest themselves on a physiological level [27,28,29]. A well-established marker providing fundamental information on the parasympathetic–sympathetic interplay is heart rate variability (HRV) (for an in-depth review see Shaffer and Ginsberg’s work [30]), which has already been applied successfully in the evaluation of physiological and emotional parameters in learning environments [15,31,32].

Other relevant internal factors, which are acquired externally from the concrete learning situation are motivational components, such as intrinsic and extrinsic motivation [33], self-determination [34,35], and self-efficacy [36,37]. While self-determination means the self-perceived degree of control and autonomy over one’s own learning success [35], self-efficacy means the perceived confidence that the respective task can be successfully completed [38]. Based on the social cognitive theory by Bandura [39,40] which identified personal and environmental factors as well as characteristics of the behavior itself as constructs affecting the outcome and emergence of certain behaviors, Glynn et al. [35] developed the Science Motivation Questionnaire II in order to holistically collect the associated factors in the context of scientific teaching.

Using the example of a practically oriented histology course in the first semester of medical studies, this investigation examined the influence of motivational factors, self-efficacy, perceived life stress, and social anxiety on HRV and learning experience in different learning environments. For this purpose, the contents of a regular practical histology course were offered in three different teaching formats; in face-to-face teaching (F2F. L), in an online transmission of the face-to-face course (passive online learning/Pas. OL.) and in an interactive online teaching unit (interactive online learning/Int. OL.), so that all constructs were measured equally in the three different learning environments. Based on previous investigations regarding physiological correlates of various teaching environments and methods [15], this in-depth analysis focuses on external factors and examines their influence on the physiological and subjective learning experience.

## 2. Materials and Methods

### 2.1. Construction of the Different Teaching Formats

The three different teaching formats were based on a regular practical 120-min course on Microscopic Anatomy in the first semester of medical school.

As part of face-to-face teaching, the students followed the learning unit in the histology lecture hall and were each equipped with a Leica DM500 microscope (Leica Microsystems GmbH, Wetzlar, Germany), with which the participants examined histological sections both through the eyepiece and on a screen connected to the microscope.

Participants engaged in passive online learning followed the pure transmission of the face-to-face course from home via zoom (Zoom Video Communications, Inc.; version 5.8.3, San Jose, CA, USA) and examined the histological sections online using the virtual microscope MyMi.mobile (Ulm University, Ulm, Germany).

In the context of interactive online learning, the participants were continuously involved in the course events using activating teaching methods [41] such as “cold call” and “random call” [42] as well as an assessment component [43] announced in advance, consisting of an online-based multiple-choice test for randomly selected students at the end of the course. This increase in interaction, based on the evidence-based active learning strategy [44,45], was accompanied by the requirement to turn on the students’ cameras to enable a more personal learning atmosphere.

### 2.2. Achievement Emotions

The achievement emotions of enjoyment, boredom, and anxiety during learning were recorded with the Achievement Emotions Questionnaire (AEQ) [11] using four items per factor on a five-point Likert scale. For the subscales of enjoyment, boredom, and anxiety, Cronbach’s alpha ranged from α = 0.85 to 0.93 [11].

### 2.3. Perceived Life Stress and Stress during the Course

Perceived life stress was assessed using the shortened 20-item version of the Perceived Stress Questionnaire (PSQ) [46], consisting of the constructs worries, tension, joy, demands, each of which was assessed using 5 items on a four-point Likert scale (for an in-depth analysis of the extended PSQ dataset over the course of three years, see Gellisch et al. [47]). Regarding their reliability, the constructs achieved Cronbach’s alpha scores ranging from α = 0.80 to 0.86 [46]. Perceived stress during the 120-min course was assessed using a visual analogue scale (VAS) [48] with ‘no stress at all’ and ‘maximum stress’ at their respective endpoints. The VAS stress was validated in advance and showed a high concordance correlation coefficient (ccc = 0.66) [49] with the Perceived Stress Scale, one of the stress-assessment gold standards developed by Cohen et al. [50].

### 2.4. Anxiety in Social Situations

Participants’ expression of anxiety in social situations was measured using the Social Interaction Anxiety Scale (SIAS) developed by Mattick and Clarke [51]. The factor was measured using 20 items on a five-point Likert scale and showed Cronbach’s alpha scores ranging from 0.88 to 0.94 among different populations [51].

### 2.5. Motivational Factors

Participants’ attitude regarding the content to be learned in the course along with internal factors was measured using the Science Motivation Questionnaire II (SMQII), developed by Glynn et al. [35]. The factors career motivation, intrinsic motivation, self-determination, self-efficacy, and grade motivation were collected with 5 items per factor using a five-point Likert scale. Cronbach’s alpha scores ranging from α = 0.81 to 0.92 [35].

### 2.6. Heart Rate Variability

HRV was measured throughout the course time in all three teaching formats using movisens ECG Move 3 sensor systems (movisens, Karlsruhe, Germany). The participants who took part in the online courses received the HRV-sensors and questionnaires with step-by-step instructions in advance to enable measurement during distance education. According to the guidelines of the Task Force of the European Society of Cardiology and the North American Society of Pacing and Electrophysiology [52], the heart rate (HR) and the parasympathetic markers SDNN (standard deviation of all RR intervals) were calculated after the data had been inspected and freed from measurement artifacts, using Kubios HRV, version 3.4.3 (Kubios Oy, Kuopio, Finland) and DataAnalyzer, version 1.13.5 (movisens GmbH, Karlsruhe, Germany).

### 2.7. Participants and Data Collection

The sample size for this study was determined through the utilization of the statistical tool G Power 3.1. Therefore, the one-way analysis of variance (ANOVA) approach was employed to ascertain the suitable sample size required for this research design. Additionally, the significance level (α) was conservatively set at 0.05 along with a large effect size of 0.4. To achieve a power of 0.9, signifying the study’s capacity to detect meaningful effects, a total sample size of 102 individuals was deemed necessary.

One hundred and one healthy first semester medical students (32 males: mean age = 20.72 ± 0.36 years; 69 females: mean age = 19.61 ± 0.18 years (mean ± SEM)) were participants of this study (Table 1). On this basis, nearly one-third of the total population of 325 first-year medical students at Ruhr University Bochum could be assessed. The group assignment was conducted randomly by the Dean’s Office of the Medical Faculty. The predefined exclusion criteria restricted study inclusion to participants without any chronic or acute mental illnesses or disorders, a history of or current dependence or abuse of alcohol or medication, as well as previous experiences of attending the Microscopic Anatomy course.

Recruitment and data collection took place at the histology lecture hall at Ruhr-University Bochum, Germany. Participants were paid for participating and gave written informed consent prior to data collection.

### 2.8. Ethical Approval and Preregistration

The study procedures were conducted in agreement with the Declaration of Helsinki and were approved by the ethics committee of the Medical Faculty at the Ruhr University Bochum (20-7135). Further, preregistration of the study procedures was conducted at the German Clinical Trials Register (DRKS-ID: DRKS00027168).

### 2.9. Statistical Analysis

Investigating associations between external factors and learning experience in the form of achievement emotions experienced directly during the course, bivariate correlation patterns were calculated using the Pearson correlation coefficient.

To ensure that the overall expression of the factors within the respective groups did not differ significantly, analyses of variance (ANOVAs) were performed, always including the teaching format (passive online learning, face-to-face learning, interactive online learning) as between-subjects factors. Therefore, heart rate variability data were checked for normality using the Shapiro–Wilk test and log-transformed if a normal distribution could not be statistically proven. The significance level was set to α = 0.05 and Bonferroni–Holm corrected *p*-values were reported for multiple testing. The effect size was indicated by partial eta squared (η^2^), and the upper and lower bounds of the 95% confidence intervals were reported.

## 3. Results

Factor expressions were described, reporting the total ratings, the mean value of each item of the factor, and the standard deviation along with the skewness (Table 2).

It could be statistically validated that the factors intrinsic motivation, career motivation, grade motivation, self-efficacy, self-determination, worries, tension, demands, and self-rated performance did not diverge significantly within the three different learning formats (*p*-values ranging from 0.08 to 0.95, η^2^ ranging from 0.001 to 0.050; see Table 3).

Significant between-group effects could be shown for the factors joy (*p* = 0.007, η^2^ = 0.096, mean = Pas. OL: 1.23, Int. OL: 1.04, F2F. L: 1.31; see Table 2 and Table 3) and SIAS (*p* = 0.016, η^2^ = 0.080, mean = Pas. OL: 0.16, Int. OL: 0.19, F2F. L: 0.15; see Table 2 and Table 3). The average heart rate was lowest in passive online learning (*p* < 0.001, η^2^ = 0.325), whereas the parasympathetic marker SDNN was highest in this teaching format (*p* < 0.001, η^2^ = 0.214).

**Figure 1:**
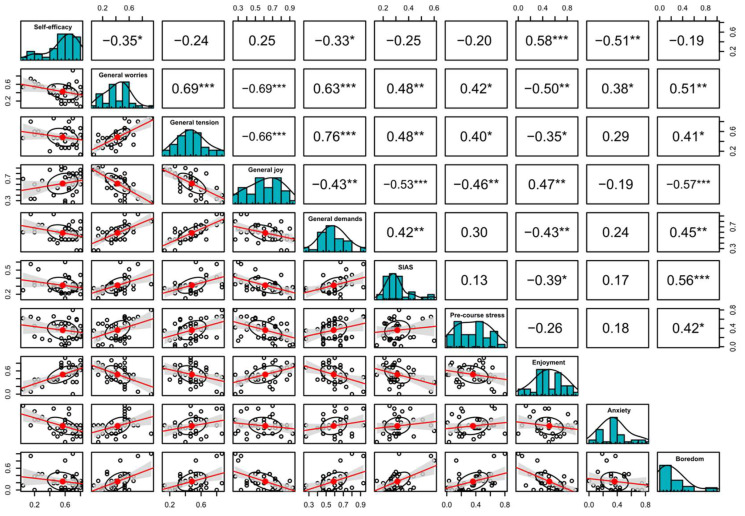
Passive online learning; a correlation matrix displaying bivariate scatter plots of the adjacent factors below the diagonal, histograms of the data distribution of the respective factors on the diagonal and the Pearson correlation above the diagonal. Ellipses specify the direction of the correlation; confidence intervals are displayed in gray. The information on the relation between two selected variables is always at right angles to each other. SIAS: Social Interaction Anxiety Scale. Ellipses indicate the direction of the correlation; confidence intervals are highlighted in gray. Asterisks indicate: * = *p* < 0.05, ** = *p* < 0.01, *** = *p* < 0.001.

**Figure 2:**
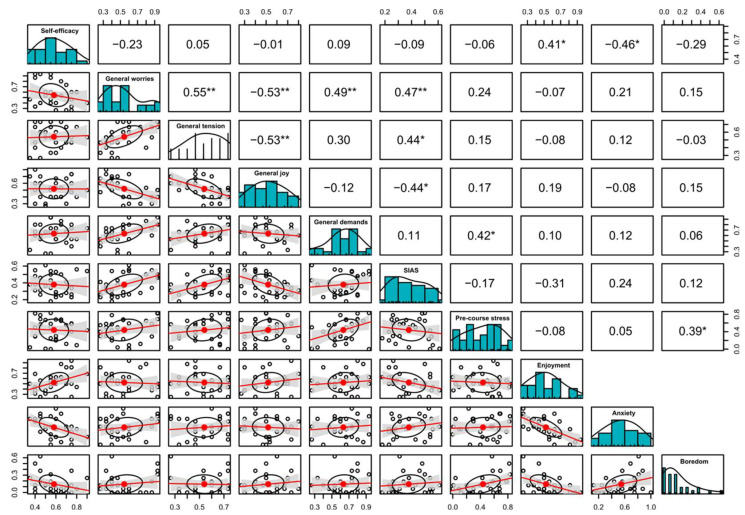
Interactive online learning; a correlation matrix displaying bivariate scatter plots of the adjacent factors below the diagonal, histograms of the data distribution of the respective factors on the diagonal and the Pearson correlation above the diagonal. Ellipses specify the direction of the correlation; confidence intervals are displayed in gray. The information on the relation between two selected variables is always at right angles to each other. SIAS: Social Interaction Anxiety Scale. Ellipses indicate the direction of the correlation; confidence intervals are highlighted in gray. Asterisks indicate: * = *p* < 0.05, ** = *p* < 0.01.

**Figure 3:**
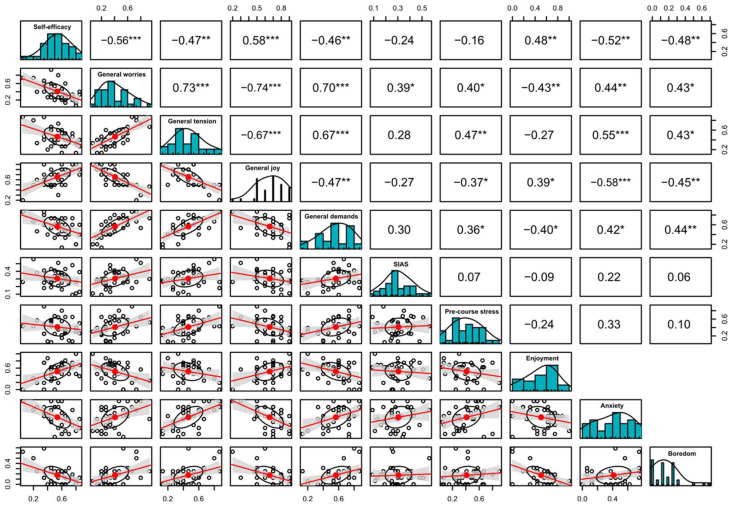
Face-to-face learning; a correlation matrix displaying bivariate scatter plots of the adjacent factors below the diagonal, histograms of the data distribution of the respective factors on the diagonal, and the Pearson correlation above the diagonal. Ellipses specify the direction of the correlation; confidence intervals are displayed in gray. The information on the relation between two selected variables is always at right angles to each other. SIAS: Social Interaction Anxiety Scale. Ellipses indicate the direction of the correlation; confidence intervals are highlighted in gray. Asterisks indicate: * = *p* < 0.05, ** = *p* < 0.01, *** = *p* < 0.001.

Significant correlations that were stable across the three different teaching formats were the positive correlation between self-efficacy and enjoyment during the course (r = Pas. OL: 0.58, Int. OL: 0.41, F2F. L: 0.48; see Figure 1, Figure 2 and Figure 3) and the negative correlation between self-efficacy and anxiety during course participation (r = Pas. OL: −0.58, Int. OL: −0.41, F2F. L: −0.48; see Figure 1, Figure 2 and Figure 3).

Other strong associations were found between general worries and anxiety during the course (r = Pas. OL: 0.38, Int. OL: 0.21, F2F. L: 0.44; see Figure 1, Figure 2 and Figure 3) and general joy of life and enjoyment during the course (r = Pas. OL: 0.47, Int. OL: 0.19, F2F. L: 0.39; see Figure 1, Figure 2 and Figure 3), along with the negative correlation between self-efficacy and boredom during course participation (r = Pas. OL: −0.19, Int. OL: −0.29, F2F. L: −0.48; see Figure 1, Figure 2 and Figure 3).

Within the cohorts of passive online learning and face-to-face learning, further negative correlations could be shown between general joy of life and boredom during the course (r = Pas. OL: −0.57, F2F. L: −0.45; see Figure 1 and Figure 3), the level of perceived life demands and enjoyment during the course (r = Pas. OL: −0.43, F2F. L: −0.40; see Figure 1 and Figure 3), and general perceived tension and enjoyment during the course (r = Pas. OL: −0.35, F2F. L: −0.27; see Figure 1 and Figure 3), along with the positive correlations of demands and tension with boredom during course participation (r = Pas. OL: 0.45, F2F. L: 0.44/Pas. OL: 0.41, F2F. L: 0.43; see Figure 1 and Figure 3). The correlations between the achievement emotions themselves are not displayed in Figure 1, Figure 2 and Figure 3 since they have already been published elsewhere [32].

**Figure 4:**
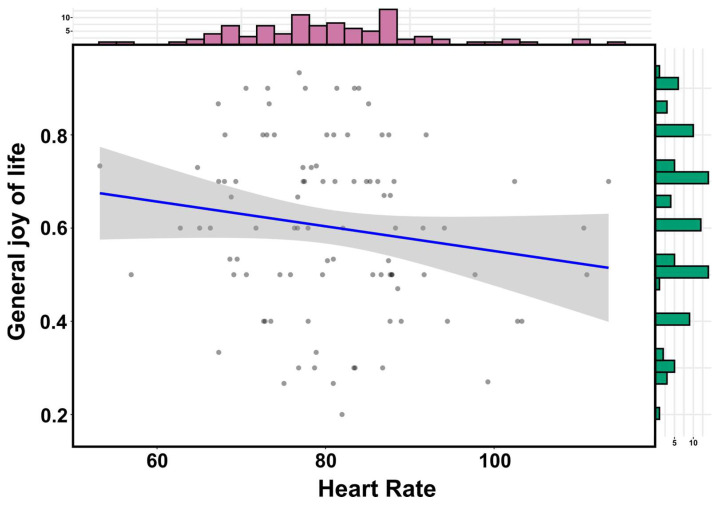
Visualization of the correlation analysis between general joy of life and heart rate during course participation (*n* = 95). The respective data distribution is visualized by bar charts. The blue line represents the line of best fit. The 95% confidence interval is highlighted in grey.

**Figure 5:**
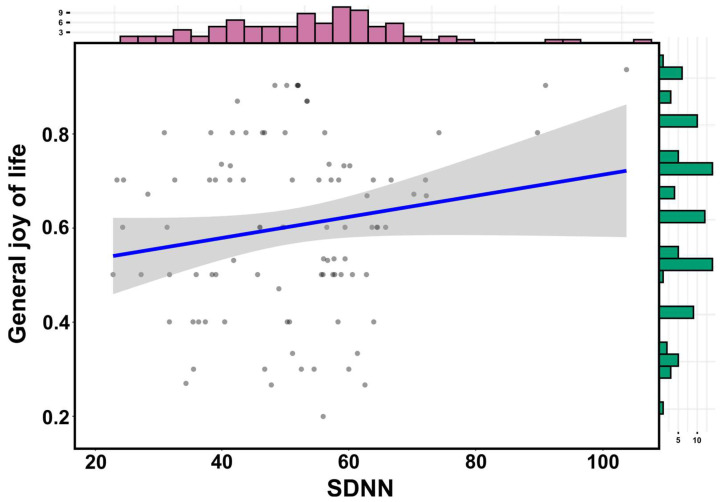
Visualization of the correlation analysis between general joy of life and the standard deviation of all RR intervals (SDNN) as a parasympathetic marker during course participation (*n* = 95). The blue line represents the line of best fit. The 95% confidence interval is highlighted in grey.

Examining the predictor qualities of external factors for the expression of physiological parameters of students during a learning unit, a negative correlation between general joy of life and heart rate (*n* = 95; r = −0.16; see Figure 4) as well as a positive correlation between general joy of life and the parasympathetic marker SDNN (*n* = 95; r = 0.18; see Figure 5) could be shown.

## 4. Discussion

Since previous research work already pointed towards a strong relationship between personal and social factors and academic achievement (for an in-depth review see Lee and Shute [53]), this study examined the relationship between external along with intrapersonal factors and immediate achievement emotions accompanied by physiological measurements during a learning unit in different learning environments.

Regardless of whether a learner participated in face-to-face learning or active or passive online learning, a consistently positive correlation between self-efficacy and enjoyment as well as a negative correlation with anxiety during the learning unit could be demonstrated. This result underpins and further explains previous research results, which demonstrated a positive correlation between self-efficacy and academic performance [54,55,56]. Similarly, this finding is contextualized by a recent study that showed that pronounced self-efficacy is associated with a lower physiological stress response in the form of decreased cortisol concentrations during a demanding cognitive task [57].

Interestingly, the findings described here additionally point to a negative correlation between self-efficacy and generally perceived worry, which supports previous research, uncovering associations between self-efficacy and various domains of human functioning with specific reference to psychopathological constructs [58]. The data described here therefore suggest that self-efficacy also constitutes a relevant construct within the population of first-year medical students that, in addition to productive dimensions in the academic context, is further characterized by protective components that can be interpreted in terms of stress-resilience.

While experimental studies regarding social anxiety have shown that a brief isolated stressor does not significantly increase subjective stress perceptions of socially anxious individuals compared to a control cohort [59,60], the data described here demonstrated that social interaction anxiety is consistently significantly correlated with the subjectively perceived stress parameter of general perceived worry in all three cohorts. On this basis, the relationship between social anxiety and long-term perceived stress can be discussed, especially in light of previously investigated interactions between stress and social anxiety along with the resulting effects on behavioral changes in terms of decision-making and risk assessment [61].

Other strong negative correlations with enjoyment during the learning unit were found between the expression of generally perceived demands and experienced tension. As these subscales contribute to the overall construct stress [46], our results complement the findings of the American College Health Association (ACHA), which demonstrated in their National College Health Assessment II, the largest known comprehensive data set on the health of college students, that 34.2% of the assessed student population experienced perceived stress as a negative influencing factor with regard to their academic performance (American College Health Association National College Health Assessment [62]). Furthermore, our results pointed towards a positive correlation between general worry and the degree of perceived anxiety during course participation, which also embeds in previous findings, demonstrating a negative correlation between worry and academic performance in 11th grade students [63]. Interestingly, the ACHA identified anxiety as the second strongest factor negatively impacting students’ academic performance, right after experienced stress [62]. On the opposite side, however, the results of this study indicate that general joy of life correlates with the positive achievement emotion of enjoyment during the learning unit. Since it could be demonstrated that positive expressions of achievement emotions predict academic achievement [8], in a more immediate manner, our results corroborate this previous research linking generally experienced positive emotions to a beneficial learning experience in academic settings [64].

Against the background that a large corpus of previous studies has already made evident that physiological arousal can have a strong influence on learning processes [65,66,67,68], special attention is paid to our result that external factors can be associated with the expression of physiological arousal during a demanding learning unit. Here, we report that general joy of life was associated with increased parasympathetic activity, which could be interpreted in terms of higher stress resilience in demanding learning situations. Further substantiating this finding, it could be shown that higher levels of general joy of life were associated with a lower heart rate during course participation, indicating attenuated sympathetic stress responses. These results provide an additional perspective on previous results demonstrating that different teaching methods and learning environments modulate students’ physiological arousal, which in turn can influence learning, learning experience, and memory processes [15,31,32,68]. However, it should be mentioned as a limitation that the observed effects of external factors in the modulation of physiological parameters during a learning unit were not sufficiently distinct that direct causality could be assumed, pointing towards the need for further validation to assess the effect size in more detail. The small effect size could possibly also lead to the conclusion that in future studies, further external factors—as physiological resilience factors—should be examined in a multivariate interaction. The object of this research project was also to address the particularly demanding situation of entering university, so that the composition of the participants was limited to first-semester students. Since it can by no means be ruled out that certain expressions of stress-associated constructs are due to the special circumstances of the entry into the academic environment, future research should investigate the effects examined in students of higher semesters. Against this background, long-term data collection would be particularly relevant, providing insight into the dynamics of students’ stress resilience during the course of their studies.

Nevertheless, this study was able to demonstrate that external factors directly influence students’ learning experience in varying learning environments.

## 5. Conclusions

While studies on the influence of external factors on educational processes have so far focused on academic performance, this study was able to demonstrate that external factors intervene much earlier in the learning process by influencing academic achievement emotions, which are closely related to learning success and experience. Thus, the results of this study were able to identify direct emotions during the learning experience as mediator variables on which external factors, such as mental health indicators, exert influence, shedding light on another operator that shapes learning success. Additionally, the results of this study also help to shed light on the constitution of physiological resilience in academic settings. Consequently, implementation of the findings of this study can occur prior to the immediate learning process by identifying relevant stressors and developing faculty-led strategies to reduce acute perceptions of stress or provide tools to strengthen stress resilience among student populations. Building on these findings, future studies should investigate external factors and relevant predictors of learning experience along with resilience in more detail and should determine more precisely at which point external factors exert their impact on learning processes to be able to offer preventive measures at an early stage. Especially at a time marked by strong social and political challenges, these results should heighten awareness regarding the relevance of aversive living conditions of students and encourage discussion of appropriate countermeasures supported by faculty.

## Figures and Tables

**Table 1:** Demographics.

	Face-to-Face-Learning	Passive Online-Learning	Interactive Online-Learning	Total
Participants	35	37	29	101
Male, *n* (%)	11 (31.43)	12 (32.43)	9 (31.03)	32 (31.68)
Female, *n* (%)	24 (68.57)	25 (67.56)	20 (68.96)	69 (68.32)
Age, mean (*SD*)	20.31 (1.98)	19.94 (1.76)	19.55 (1.19)	19.96 (1.73)
Complete questionnaire data set, *n* (%)	35 (100)	37 (100)	29 (100)	101 (100)
Complete HRV data set, *n* (%)	33 (94.28)	35 (94.59)	27 (93.10)	95 (94.06)

Note. HRV = heart rate variability.

**Table 2:** Factor description.

Factors	*n*	Rating			Mean			SD			Skewness		
		Pas. OL	Int. OL	F2F. L	Pas. OL	Int. OL	F2F. L	Pas. OL	Int. OL	F2F. L	Pas. OL	Int. OL	F2F. L.
Enjoyment	4	5.08	5.24	5.05	1.27	1.31	1.26	0.22	0.19	0.25	−0.26	0.37	−0.45
Anxiety	4	3.61	5.45	4.13	0.90	1.36	1.03	0.19	0.23	0.22	0.55	−0.10	−0.23
Boredom	4	2.35	1.44	1.75	0.59	0.36	0.44	0.25	0.16	0.17	1.54	1.47	1.39
Mental load	6	6.74	6.38	6.81	1.12	1.06	1.14	0.12	0.14	0.15	0.15	−0.03	−0.30
Mental effort	6	8.29	8.89	8.67	1.38	1.48	1.45	0.16	0.14	0.12	−0.85	−1.45	−0.89
Intrinsic motivation	5	5.59	5.48	5.01	1.12	1.10	1.00	0.20	0.15	0.19	−0.03	−0.13	−0.47
Grade motivation	5	6.43	5.78	5.94	1.29	1.16	1.19	0.26	0.25	0.20	−0.62	−0.13	−0.33
Career motivation	5	5.26	5.21	4.24	1.05	1.04	0.85	0.23	0.19	0.22	0.15	−0.01	0.21
Self determination	5	5.78	5.64	5.79	1.12	1.13	1.16	0.28	0.18	0.16	−0.40	−0.18	−0.49
Self-efficacy	5	5.68	5.81	5.37	1.14	1.16	1.07	0.20	0.14	0.18	−1.06	0.30	−0.41
Social Interaction Anxiety Scale	20	3.11	3.79	3.05	0.16	0.19	0.15	0.10	0.12	0.11	1.36	0.25	0.18

Note. *n* = number of items per construct, Rating = overall factor ratings, Mean = mean value of each item of the factor, SD = standard deviation, Pas. OL = passive online learning, Int. OL = interactive online learning, F2F. L = face-to-face learning.

**Table 3:** ANOVA and Post-hoc analyses.

Factors	ANOVA			Post-hoc Analyses		
				Group 1–2	Group 1–3	Group 2–3
	F	*p*	η^2^	*p* [95% Cl–lower and upper bound]		
Intrinsic motivation	0.984	0.38	0.020	0.55 [−0.045, 0.161]	0.81 [−0.097, 0.120]	0.63 [−0.063, 0.157]
Career motivation	2.419	0.09	0.047	0.15 [−0.020, 0.222]	0.93 [−0.122, 0.132]	0.16 [−0.033, 0.225]
Grade Motivation	0.667	0.51	0.013	0.84 [−0.088, 0.186]	0.84 [−0.078, 0.210]	0.84 [−0.162, 0.129]
Self-efficacy	0.518	0.59	0.010	0.99 [−0.070, 0.131]	0.99 [−0.119, 0.092]	0.99 [−0.063, 0.151]
Self-determination	0.046	0.95	0.001	1 [−0.123, 0.123]	1 [−0.114, 0.144]	1 [−0.146, 0.116]
Worries	2.561	0.08	0.050	0.84 [−0.140, 0.118]	0.12 [−0.256, 0.016]	0.13 [−0.029, 0.246]
Tension	1.609	0.20	0.032	0.66 [−0.079, 0.115]	0.37 [−0.159, 0.045]	0.26 [−0.028, 0.179]
Demands	1.356	0.26	0.027	0.56 [−0.073, 0.120]	0.55 [−0.140, 0.055]	0.32 [−0.032, 0.174]
Joy	5.214	0.007	0.096	0.315 [−0.138, 0.056]	0.053 [−0.005, 0.198]	0.006 [−0.241, −0.034]
Perceived Stress Questionnaire (total)	3.661	0.003	0.070	0.583 [−0.088, 0.140]	0.078 [−0.225, 0.014]	0.034 [0.010, 0.253]
Social Interaction Anxiety Scale	4.289	0.016	0.080	0.80 [−0.056, 0.069]	0.03 [−0.133, −0.003]	0.026 [0.008, 0.141]
Preference Face-to-face learning	2.106	0.12	0.041	0.15 [−0.019, 0.208]	0.35 [−0.050, 0.188]	0.61 [−0.095, 0.146]
Self-rated performance	0.26	0.76	0.005	1 [−0.081, 0.137]	1 [−0.117, 0.112]	1 [−0.085, 0.147]
Heart Rate	22.12	<0.001	0.325	<0.001 [−0.091, −0.034]	<0.001 [−0.106, −0.046]	0.29 [−0.017, 0.044]
SDNN	12.55	<0.001	0.214	<0.001 [0.050, 0.183]	<0.001 [0.059, 0.199]	0.686 [−0.083, 0.059]

Note. Group 1 = passive online learning, Group 2 = face-to-face learning, Group 3 = interactive online learning: F = F-value, *p* = *p*-value, η^2^ = partial eta squared, CI = confidence intervals, SDNN = standard deviation of the NN intervals.

## Data Availability

The data presented in this study are available on request from the corresponding author.

## References

[1] Chickering A.W., Gamson Z.F. (1991). Appendix A: Seven Principles for Good Practice in Undergraduate Education. New Dir. Teach. Learn..

[2] Remedios R., Lieberman D.A. (2008). I Liked Your Course Because You Taught Me Well: The Influence of Grades, Workload, Expectations and Goals on Students’ Evaluations of Teaching. Br. Educ. Res. J..

[3] Reschly A.L., Christenson S.L. (2012). Jingle, Jangle, and Conceptual Haziness: Evolution and Future Directions of the Engagement Construct. Handbook of Research on Student Engagement.

[4] Nolen S.B., Horn I.S., Ward C.J. (2015). Situating Motivation. Educ. Psychol..

[5] Hiver P., Zhou S.A., Tahmouresi S., Sang Y., Papi M. (2020). Why Stories Matter: Exploring Learner Engagement and Metacognition through Narratives of the L2 Learning Experience. System.

[6] Pekrun R. (2007). Emotions in Students’ Scholastic Development. The Scholarship of Teaching and Learning in Higher Education: An Evidence-Based Perspective.

[7] Mega C., Ronconi L., de Beni R. (2014). What Makes a Good Student? How Emotions, Self-Regulated Learning, and Motivation Contribute to Academic Achievement. J. Educ. Psychol..

[8] Pekrun R., Lichtenfeld S., Marsh H.W., Murayama K., Goetz T. (2017). Achievement Emotions and Academic Performance: Longitudinal Models of Reciprocal Effects. Child. Dev..

[9] Camacho-Morles J., Slemp G.R., Pekrun R., Loderer K., Hou H., Oades L.G. (2021). Activity Achievement Emotions and Academic Performance: A Meta-Analysis. Educ. Psychol. Rev..

[10] Carmona-Halty M., Salanova M., Llorens S., Schaufeli W.B. (2021). Linking Positive Emotions and Academic Performance: The Mediated Role of Academic Psychological Capital and Academic Engagement. Curr. Psychol..

[11] Pekrun R., Goetz T., Frenzel A.C., Barchfeld P., Perry R.P. (2011). Measuring Emotions in Students’ Learning and Performance: The Achievement Emotions Questionnaire (AEQ). Contemp. Educ. Psychol..

[12] Pekrun R., Goetz T., Titz W., Perry R.P. (2002). Academic Emotions in Students’ Self-Regulated Learning and Achievement: A Program of Qualitative and Quantitative Research. Educ. Psychol..

[13] Lumpkin A., Achen R.M., Dodd R.K. (2015). Student Perceptions of Active Learning. Coll. Stud. J..

[14] Jeong J.S., González-Gómez D., Cañada-Cañada F., Gallego-Picó A., Bravo J.C. (2019). Effects of Active Learning Methodologies on the Students’ Emotions, Self-Efficacy Beliefs and Learning Outcomes in a Science Distance Learning Course. J. Technol. Sci. Educ..

[15] Gellisch M., Morosan-Puopolo G., Wolf O.T., Moser D.A., Zaehres H., Brand-Saberi B. (2023). Interactive Teaching Enhances Students’ Physiological Arousal during Online Learning. Ann. Anat. Anat. Anz..

[16] Cahill J., Turner J., Barefoot H. (2010). Enhancing the Student Learning Experience: The Perspective of Academic Staff. Educ. Res..

[17] Ragpala E. (2021). Environmental Factors That Affect the Academic Performance of Senior High School Students during COVID-19 Pandemic. Int. J. Social. Sci. Curr. Future Res. Trends.

[18] Hammen C. (2005). Stress and Depression. Annu. Rev. Clin. Psychol..

[19] Reiche E.M.V., Morimoto H.K., Nunes S.M.V. (2005). Stress and Depression-Induced Immune Dysfunction: Implications for the Development and Progression of Cancer. Int. Rev. Psychiatry.

[20] Nielsen N.R., Kristensen T.S., Schnohr P., Gronbaek M. (2008). Perceived Stress and Cause-Specific Mortality among Men and Women: Results from a Prospective Cohort Study. Am. J. Epidemiol..

[21] Wiegner L., Hange D., Björkelund C., Ahlborg G. (2015). Prevalence of Perceived Stress and Associations to Symptoms of Exhaustion, Depression and Anxiety in a Working Age Population Seeking Primary Care—An Observational Study. BMC Fam. Pract..

[22] Crozier R. (2000). Shyness.

[23] Veale D. (2003). Treatment of Social Phobia. Adv. Psychiatr. Treat..

[24] Russell G., Topham P. (2012). The Impact of Social Anxiety on Student Learning and Well-Being in Higher Education. J. Ment. Health.

[25] Bernstein G.A., Bernat D.H., Davis A.A., Layne A.E. (2008). Symptom Presentation and Classroom Functioning in a Nonclinical Sample of Children with Social Phobia. Depress. Anxiety.

[26] Leigh E., Chiu K., Clark D.M. (2021). Is Concentration an Indirect Link between Social Anxiety and Educational Achievement in Adolescents?. PLoS ONE.

[27] Kirschbaum C., Pirke K.-M., Hellhammer D.H. (1993). The ‘Trier Social Stress Test’—A Tool for Investigating Psychobiological Stress Responses in a Laboratory Setting. Neuropsychobiology.

[28] Kaczmarek L.D., Behnke M., Enko J., Kosakowski M., Hughes B.M., Piskorski J., Guzik P. (2019). Effects of Emotions on Heart Rate Asymmetry. Psychophysiology.

[29] Folz J., Fiacchino D., Nikolić M., van Steenbergen H., Kret M.E. (2022). Reading Your Emotions in My Physiology? Reliable Emotion Interpretations in Absence of a Robust Physiological Resonance. Affect. Sci..

[30] Shaffer F., Ginsberg J.P. (2017). An Overview of Heart Rate Variability Metrics and Norms. Front. Public. Health.

[31] Minkley N., Ringeisen T., Josek L.B., Kärner T. (2017). Stress and Emotions during Experiments in Biology Classes: Does the Work Setting Matter?. Contemp. Educ. Psychol..

[32] Gellisch M., Wolf O.T., Minkley N., Kirchner W.H., Brüne M., Brand-Saberi B. (2022). Decreased Sympathetic Cardiovascular Influences and Hormone-physiological Changes in Response to Covid-19-related Adaptations under Different Learning Environments. Anat. Sci. Educ..

[33] Pintrich P.R. (2003). A Motivational Science Perspective on the Role of Student Motivation in Learning and Teaching Contexts. J. Educ. Psychol..

[34] Black A.E., Deci E.L. (2000). The Effects of Instructors’ Autonomy Support and Students’ Autonomous Motivation on Learning Organic Chemistry: A Self-Determination Theory Perspective. Sci. Educ..

[35] Glynn S.M., Brickman P., Armstrong N., Taasoobshirazi G. (2011). Science Motivation Questionnaire II: Validation with Science Majors and Nonscience Majors. J. Res. Sci. Teach..

[36] Zimmerman B.J. (2000). Self-Efficacy: An Essential Motive to Learn. Contemp. Educ. Psychol..

[37] Lawson A.E., Banks D.L., Logvin M. (2007). Self-Efficacy, Reasoning Ability, and Achievement in College Biology. J. Res. Sci. Teach..

[38] Bandura A., Freeman W.H., Lightsey R. (1999). Self-Efficacy: The Exercise of Control. J. Cogn. Psychother..

[39] Bandura A. (1986). Social Foundations of Thought and Action.

[40] Bandura A. (1999). Social Cognitive Theory: An Agentic Perspective. Asian J. Soc. Psychol..

[41] Waltz C.F., Jenkins L.S., Han N. (2014). The Use and Effectiveness of Active Learning Methods in Nursing and Health Professions Education: A Literature Review. Nurs. Educ. Perspect..

[42] Cooper K.M., Downing V.R., Brownell S.E. (2018). The Influence of Active Learning Practices on Student Anxiety in Large-Enrollment College Science Classrooms. Int. J. STEM Educ..

[43] Freeman S., Eddy S.L., McDonough M., Smith M.K., Okoroafor N., Jordt H., Wenderoth M.P. (2014). Active Learning Increases Student Performance in Science, Engineering, and Mathematics. Proc. Natl. Acad. Sci. USA.

[44] Dallimore E.J., Hertenstein J.H., Platt M.B. (2013). Impact of Cold-Calling on Student Voluntary Participation. J. Manag. Educ..

[45] Eddy S.L., Converse M., Wenderoth M.P. (2015). PORTAAL: A Classroom Observation Tool Assessing Evidence-Based Teaching Practices for Active Learning in Large Science, Technology, Engineering, and Mathematics Classes. CBE—Life Sci. Educ..

[46] Fliege H., Rose M., Arck P., Levenstein S., Klapp B.F. (2001). Validierung Des “Perceived Stress Questionnaire“ (PSQ) an Einer Deutschen Stichprobe. Diagnostica.

[47] Gellisch M., Bablok M., Morosan-Puopolo G., Schäfer T., Brand-Saberi B. (2023). Dynamically Changing Mental Stress Parameters of First-Year Medical Students over the Three-Year Course of the COVID-19 Pandemic: A Repeated Cross-Sectional Study. Healthcare.

[48] Luria R.E. (1975). The Validity and Reliability of the Visual Analogue Mood Scale. J. Psychiatr. Res..

[49] Lesage F.X., Berjot S. (2011). Validity of Occupational Stress Assessment Using a Visual Analogue Scale. Occup. Med. (Chic III).

[50] Cohen S., Kamarck T., Mermelstein R. (1983). A Global Measure of Perceived Stress. J. Health Soc. Behav..

[51] Mattick R.P., Clarke J.C. (1998). Development and Validation of Measures of Social Phobia Scrutiny Fear and Social Interaction Anxiety. Behav. Res. Ther..

[52] Malik M. (1996). Heart Rate Variability—Standards of Measurement, Physiological Interpretation, and Clinical Use: Task Force of The European Society of Cardiology and the North American Society for Pacing and Electrophysiology. Ann. Noninvasive Electrocardiol..

[53] LEE J., SHUTE V.J. (2010). Personal and Social-Contextual Factors in K–12 Academic Performance: An Integrative Perspective on Student Learning. Educ. Psychol..

[54] Chemers M.M., Hu L., Garcia B.F. (2001). Academic Self-Efficacy and First Year College Student Performance and Adjustment. J. Educ. Psychol..

[55] Burgoon J.M., Meece J.L., Granger N.A. (2012). Self-Efficacy’s Influence on Student Academic Achievement in the Medical Anatomy Curriculum. Anat. Sci. Educ..

[56] Vogel F.R., Human-Vogel S. (2016). Academic Commitment and Self-Efficacy as Predictors of Academic Achievement in Additional Materials Science. High. Educ. Res. Dev..

[57] Schickel M., Minkley N., Ringeisen T. (2023). Performance during Presentations: A Question of Challenge and Threat Responses?. Contemp. Educ. Psychol..

[58] Luszczynska A., Gutiérrez-Doña B., Schwarzer R. (2005). General Self-efficacy in Various Domains of Human Functioning: Evidence from Five Countries. Int. J. Psychol..

[59] Shirotsuki K., Izawa S., Sugaya N., Yamada K.C., Ogawa N., Ouchi Y., Nagano Y., Nomura S. (2009). Salivary Cortisol and DHEA Reactivity to Psychosocial Stress in Socially Anxious Males. Int. J. Psychophysiol..

[60] Condren R. (2002). HPA Axis Response to a Psychological Stressor in Generalised Social Phobia. Psychoneuroendocrinology.

[61] Hengen K.M., Alpers G.W. (2021). Stress Makes the Difference: Social Stress and Social Anxiety in Decision-Making Under Uncertainty. Front. Psychol..

[62] American College Health Association (ACHA) (2019). National College Health Assessment II.

[63] Steinmayr R., Crede J., McElvany N., Wirthwein L. (2016). Subjective Well-Being, Test Anxiety, Academic Achievement: Testing for Reciprocal Effects. Front. Psychol..

[64] Rodríguez-Muñoz A., Antino M., Ruiz-Zorrilla P., Ortega E. (2021). Positive Emotions, Engagement, and Objective Academic Performance: A Weekly Diary Study. Learn. Individ. Differ..

[65] Joëls M., Pu Z., Wiegert O., Oitzl M.S., Krugers H.J. (2006). Learning under Stress: How Does It Work?. Trends Cogn. Sci..

[66] Roozendaal B., McGaugh J.L. (2011). Memory Modulation. Behav. Neurosci..

[67] Sapolsky R.M. (2015). Stress and the Brain: Individual Variability and the Inverted-U. Nat. Neurosci..

[68] Wolf O.T. (2017). Stress and Memory Retrieval: Mechanisms and Consequences. Curr. Opin. Behav. Sci..

